# Pyridinium 3-nitro­benzoate–3-nitro­benzoic acid (1/1)

**DOI:** 10.1107/S2414314621005812

**Published:** 2021-06-15

**Authors:** Alexis Howarth, Tony J. Barbosa, Matthias Zeller, Patrick C. Hillesheim

**Affiliations:** a Ave Maria University, Department of Chemistry and Physics, 5050 Ave Maria Blvd, Ave Maria FL, 34142, USA; b Purdue University, Department of Chemistry, 560 Oval Drive, West Lafayette, Indiana USA, 47907, USA; Benemérita Universidad Autónoma de Puebla, México

**Keywords:** crystal structure, hydrogen bonding, carboxyl­ate, carb­oxy­lic acid

## Abstract

The crystal structure of the product of the neutralization reaction between 3-nitro­benzoic acid and pyridine is reported. The entities that crystallized are a pyridinium cation, a 3-nitrobenzoate anion and a 3-nitrobenzoic acid molecule in a 1:1:1 molar ratio, C_5_H_6_N^+^·C_7_H_4_NO_4_^−^·C_7_H_5_NO_4_. Distinct sets of hydrogen bonds link the pyridinium and benzoate ions (N—H⋯O) and the acid and benzoate moieties (O—H⋯O). The hydrogen bonding along with π–π stacking between the acid and benzoate moieties accounts for the long-range ordering of the crystal.

## Abstract 2

The structure of the neutralization product of 3-nitro­benzoic acid and pyridine is reported. A combination of hydrogen bonding and π–π inter­actions link the mol­ecules, forming long-range order.

## Graphical abstract

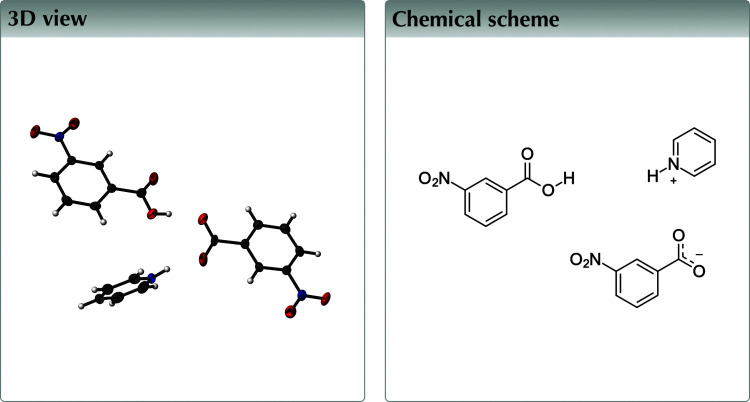

## Structure description

**Figure 1:**
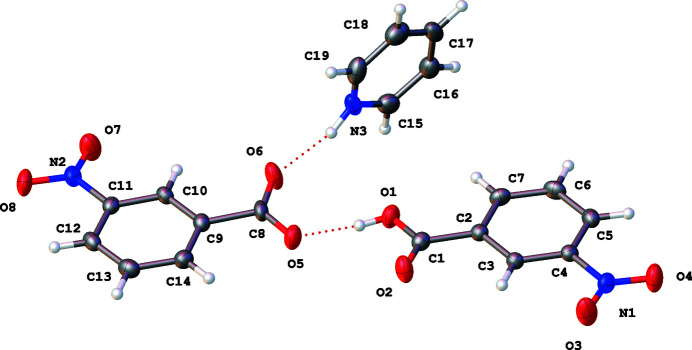
The asymmetric unit of the structure with 50% probability ellipsoids and hydrogen bonds indicated by red dotted lines.

The sample crystallizes in the monoclinic crystal system in the *Pc* space group. Three discrete entities in a 1:1:1 molar ratio comprise the asymmetric unit of this structure: 3-nitro­benzoic acid, 3-nitro­benzoate, and a pyridinium cation (Fig. 1 ▸). The structure is the result of a neutralization reaction of the carb­oxy­lic acid and pyridine (see *Synthesis and crystallization* section for details). The benzoic acid molecule and benzoate anion in the asymmetric unit are nearly coplanar with a 1.16 (14)° dihedral angle. The dihedral angle between the pyridinium and the acid is 99.99 (10)° and the dihedral angle between the benzoate anion and pyridinium cation is 99.58 (10)°.

The acid and benzoate moieties are linked through a short hydrogen bond between the protonated carb­oxy­lic acid oxygen atom O1 and the carboxyl­ate anion oxygen Oatom 5 [O1—H1⋯O5, *d* = 1.69 (4) Å]. The other carboxyl­ate oxygen atom, O6, accepts a hydrogen bond from the protonated pyridinium cation, N3—H3*A*⋯O6 at a distance of 1.81 (4) Å (Figs. 1 ▸ and 2 ▸; Table 1 ▸).

**Figure 3:**
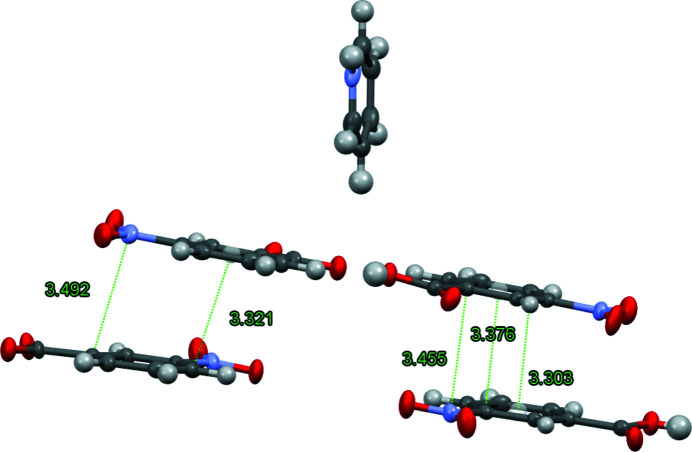
Depiction of the π inter­actions in the title structure. Lines in green display multiple close points of contact between the aromatic rings. Distances in Å.

Parallel, offset π inter­actions between benzoate anions and between benzoic acid molecules account, in part, for the long-range ordering of the structure. The inter­actions range in distance from approximately 3.3 to 3.5 Å. Given the offset inter­actions of the aromatic rings, it appears that inter­actions are between the nitro groups and the aromatic rings in a manner similar to previously reported structures (Sánchez-Moreno *et al.*, 2003 ▸). A depiction of these π inter­actions is shown in Fig. 3 ▸. No π inter­actions are observed from the pyridinium moiety.

Both distinct nitro groups, that is the nitro group on the acid molecule and the nitro group on the benzoate anion, inter­act with hydrogen atoms on the pyridinium ring. The shortest H⋯O_NO2_ inter­actions are between O7 and O8 with H19 in one of the α positions of the pyridinium ring. Both O atoms of the nitro moiety participate in a nearly symmetric, bifurcated inter­action with the H19 atom at distances of 2.695 (3) and 2.714 (3) Å, respectively. The other nitro oxygen atoms (O3 and O4) also display a nearly symmetric bifurcated set of inter­actions with H16 in the β position of the pyridinium ring, at H⋯O_NO2_ distances of 2.882 (3) and 2.820 (3) Å, respectively. The H⋯O_NO2_ inter­actions observed herein are similar to those observed in some previously reported compounds (Allen *et al.*, 1997 ▸; Gu *et al.*, 1999 ▸; Vijayvergiya *et al.*, 1995 ▸).

## Synthesis and crystallization

The reported crystal is an impurity from residual water from an esterification reaction. A sample of 3-nitro­benzoyl chloride (1 eq.) was dissolved in di­chloro­methane (30 ml) with stirring. Pyridine (2 eq.) and ethanol (5 eq.) were added to the solution, the flask sealed, and the entire mixture allowed to stir overnight at room temperature. A white crystalline solid formed after several minutes of stirring. A sample of this crystalline material was collected and analyzed, yielding the structure presented herein.

## Refinement

Crystal data, data collection and structure refinement details are summarized in Table 2 ▸. The spatial arrangement of the two nitro­aromatic moieties in the asymmetric unit, with the exception of the acidic proton on the carbonyl group forming the acid *versus* the carboxyl­ate group, might lead to the conclusion that a higher crystallographic symmetry would exist. As such, these two mol­ecules appear related by pseudo screw-axis symmetry; however, they are, in fact, not related by symmetry. To verify this claim, the structure was solved in the *P*2_1_/*c* space group, which leads to a substantial increase in the *R*_1_ and *wR*_2_ residuals (11.78% and 23.88%, respectively). The final structure solution presented is thus in the correct space group, accounting for the subtle differences in the bonding of two nitro­aromatic moieties.

## Supplementary Material

Crystal structure: contains datablock(s) I. DOI: 10.1107/S2414314621005812/bh4062sup1.cif

Structure factors: contains datablock(s) I. DOI: 10.1107/S2414314621005812/bh4062Isup3.hkl

Supporting information file. DOI: 10.1107/S2414314621005812/bh4062Isup3.cml

CCDC reference: 2088013

Additional supporting information:  crystallographic information; 3D view; checkCIF report

## Figures and Tables

**Figure 2:**
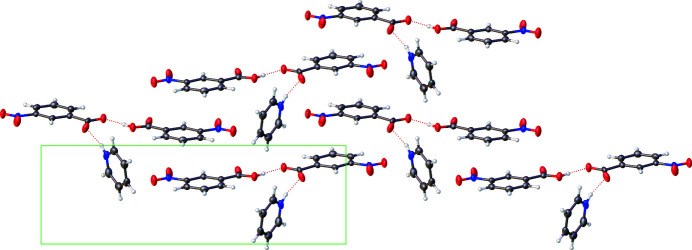
Packing diagram of the title compound viewed from the (100) face.

**Table 1:** Hydrogen-bond geometry (Å, °)

*D*—H⋯*A*	*D*—H	H⋯*A*	*D*⋯*A*	*D*—H⋯*A*
O1—H1⋯O5	0.83 (4)	1.69 (4)	2.508 (3)	169 (5)
N3—H3*A*⋯O6	0.88 (4)	1.81 (4)	2.667 (4)	164 (4)

**Table 2:** Experimental details

Crystal data	
Chemical formula	C_5_H_6_N^+^·C_7_H_4_NO_4_^−^·C_7_H_5_NO_4_
*M*_r_	413.34
Crystal system, space group	Monoclinic, *P* *c*
Temperature (K)	150
*a*, *b*, *c* (Å)	6.2434 (3), 21.3584 (10), 6.8938 (3)
β (°)	93.118 (2)
*V* (Å^3^)	917.92 (7)
*Z*	2
Radiation type	Mo *K*α
μ (mm^−1^)	0.12
Crystal size (mm)	0.35 × 0.15 × 0.05

Data collection	
Diffractometer	Bruker AXS D8 Quest diffractometer with PhotonII charge-integrating pixel array detector (CPAD)
Absorption correction	Multi-scan (*SADABS*; Bruker, 2020 ▸)
*T*_min_, *T*_max_	0.636, 0.746
No. of measured, independent and observed [*I* > 2σ(*I*)] reflections	7895, 4437, 3733
*R*_int_	0.023
(sin θ/λ)_max_ (Å^−1^)	0.669

Refinement	
*R*[*F* ^2^ > 2σ(*F* ^2^)], *wR*(*F* ^2^), *S*	0.044, 0.107, 1.05
No. of reflections	4437
No. of parameters	277
No. of restraints	2
H-atom treatment	H atoms treated by a mixture of independent and constrained refinement
Δρ_max_, Δρ_min_ (e Å^−3^)	0.25, −0.19

Computer programs: *APEX3* (Bruker, 2020 ▸), *SAINT* (Bruker, 2020 ▸), *SHELXT2018/2* (Sheldrick, 2015*a* ▸), *SHELXL2018/3* (Sheldrick, 2015*b* ▸), *OLEX2* (Dolomanov *et al.*, 2009 ▸); *Mercury* (Macrae *et al.*, 2020 ▸), *publCIF* (Westrip, 2010 ▸), CSD (Groom *et al.*, 2016 ▸) and *enCIFer* (Allen *et al.*, 2004 ▸).

## full crystallographic data

### Crystal data

**Table:**

C_5_H_6_N^+^·C_7_H_4_NO_4_^−^·C_7_H_5_NO_4_	*F*(000) = 428
*M**_r_* = 413.34	*D*_x_ = 1.495 Mg m^−^^3^
Monoclinic, *P**c*	Mo *K*α radiation, λ = 0.71073 Å
*a* = 6.2434 (3) Å	Cell parameters from 3891 reflections
*b* = 21.3584 (10) Å	θ = 3.0–28.3°
*c* = 6.8938 (3) Å	µ = 0.12 mm^−^^1^
β = 93.118 (2)°	*T* = 150 K
*V* = 917.92 (7) Å^3^	Plate, colourless
*Z* = 2	0.35 × 0.15 × 0.05 mm

### Data collection

**Table:**

Bruker AXS D8 Quest diffractometer with PhotonII charge-integrating pixel array detector (CPAD)	4437 independent reflections
Radiation source: 1 kW fine focus sealed tube X-ray source	3733 reflections with *I* > 2σ(*I*)
Detector resolution: 7.4074 pixels mm^-1^	*R*_int_ = 0.023
ω and φ scans	θ_max_ = 28.4°, θ_min_ = 2.9°
Absorption correction: multi-scan (SADABS; Bruker, 2020)	*h* = −8→8
*T*_min_ = 0.636, *T*_max_ = 0.746	*k* = −28→28
7895 measured reflections	*l* = −9→9

### Refinement

**Table:**

Refinement on *F*^2^	Primary atom site location: dual
Least-squares matrix: full	Secondary atom site location: difference Fourier map
*R*[*F*^2^ > 2σ(*F*^2^)] = 0.044	Hydrogen site location: mixed
*wR*(*F*^2^) = 0.107	H atoms treated by a mixture of independent and constrained refinement
*S* = 1.05	*w* = 1/[σ^2^(*F*_o_^2^) + (0.044*P*)^2^ + 0.2733*P*] where *P* = (*F*_o_^2^ + 2*F*_c_^2^)/3
4437 reflections	(Δ/σ)_max_ < 0.001
277 parameters	Δρ_max_ = 0.25 e Å^−^^3^
2 restraints	Δρ_min_ = −0.19 e Å^−^^3^

### Special details

**Table:**

Refinement. H atoms on the aromatic (*sp*^2^) carbons were included in calculated positions and treated as riding atoms: C—H = 0.95 Å with *U*_iso_(H) = 1.2 × *U*_eq_(carrier atom). H atoms H1 and H3A were located as residual electron density and allowed to refine freely with *U*_iso_(H) = 1.5 × *U*_eq_(O or N).

### Fractional atomic coordinates and isotropic or equivalent isotropic displacement parameters (Å^2^)

**Table:**

	*x*	*y*	*z*	*U*_iso_*/*U*_eq_	
C1	0.3880 (5)	0.35185 (15)	0.2998 (4)	0.0229 (7)	
C2	0.5147 (5)	0.40980 (15)	0.3404 (4)	0.0203 (7)	
C3	0.4093 (5)	0.46720 (14)	0.3147 (4)	0.0190 (7)	
H3	0.262180	0.468543	0.271691	0.023*	
C4	0.5217 (5)	0.52186 (15)	0.3528 (4)	0.0198 (7)	
C5	0.7352 (5)	0.52249 (16)	0.4169 (5)	0.0241 (7)	
H5	0.809070	0.560736	0.442560	0.029*	
C6	0.8384 (5)	0.46494 (16)	0.4425 (5)	0.0261 (7)	
H6	0.985232	0.463902	0.486611	0.031*	
C7	0.7308 (5)	0.40954 (16)	0.4048 (5)	0.0235 (7)	
H7	0.804206	0.370838	0.422786	0.028*	
N1	0.4075 (5)	0.58181 (13)	0.3261 (4)	0.0254 (6)	
O1	0.5027 (4)	0.30049 (11)	0.3136 (4)	0.0325 (6)	
H1	0.419 (7)	0.2717 (19)	0.283 (7)	0.049*	
O2	0.1965 (4)	0.35353 (11)	0.2591 (4)	0.0346 (6)	
O3	0.2159 (4)	0.58001 (12)	0.2787 (5)	0.0421 (7)	
O4	0.5076 (5)	0.63004 (12)	0.3523 (4)	0.0410 (7)	
C8	0.3862 (6)	0.15289 (15)	0.2486 (5)	0.0246 (7)	
C9	0.2590 (5)	0.09406 (15)	0.1956 (4)	0.0213 (7)	
C10	0.3561 (6)	0.03643 (14)	0.2200 (4)	0.0208 (7)	
H10	0.501855	0.033298	0.266289	0.025*	
C11	0.2370 (5)	−0.01667 (14)	0.1758 (4)	0.0213 (7)	
C12	0.0235 (5)	−0.01458 (16)	0.1095 (4)	0.0239 (7)	
H12	−0.055428	−0.051872	0.082220	0.029*	
C13	−0.0699 (6)	0.04316 (16)	0.0848 (5)	0.0275 (8)	
H13	−0.215401	0.046060	0.037739	0.033*	
C14	0.0457 (6)	0.09765 (16)	0.1278 (5)	0.0266 (7)	
H14	−0.021382	0.137334	0.110787	0.032*	
N2	0.3439 (5)	−0.07747 (13)	0.2017 (4)	0.0278 (7)	
O5	0.2900 (4)	0.20430 (11)	0.2221 (4)	0.0343 (6)	
O6	0.5729 (4)	0.14586 (12)	0.3167 (4)	0.0366 (6)	
O7	0.5346 (5)	−0.07837 (13)	0.2492 (5)	0.0448 (7)	
O8	0.2350 (5)	−0.12516 (12)	0.1741 (4)	0.0376 (6)	
C15	0.9530 (6)	0.23917 (17)	0.5534 (5)	0.0337 (8)	
H15	1.002933	0.233689	0.426833	0.040*	
C16	1.0720 (6)	0.27288 (17)	0.6888 (6)	0.0337 (8)	
H16	1.204965	0.290825	0.657419	0.040*	
C17	0.9967 (6)	0.28047 (15)	0.8709 (5)	0.0352 (8)	
H17	1.078436	0.303379	0.967064	0.042*	
C18	0.8023 (7)	0.25478 (17)	0.9138 (6)	0.0392 (9)	
H18	0.747524	0.260163	1.038592	0.047*	
C19	0.6901 (7)	0.22142 (16)	0.7730 (6)	0.0372 (8)	
H19	0.555815	0.203374	0.799899	0.045*	
N3	0.7677 (5)	0.21406 (13)	0.5984 (5)	0.0321 (7)	
H3A	0.694 (7)	0.198 (2)	0.498 (6)	0.048*	

### Atomic displacement parameters (Å^2^)

**Table:**

	*U*^11^	*U*^22^	*U*^33^	*U*^12^	*U*^13^	*U*^23^
C1	0.0250 (17)	0.0217 (15)	0.0224 (15)	0.0023 (13)	0.0047 (13)	0.0001 (12)
C2	0.0209 (16)	0.0212 (15)	0.0189 (15)	0.0001 (12)	0.0026 (12)	0.0000 (11)
C3	0.0177 (17)	0.0205 (15)	0.0188 (15)	0.0009 (12)	0.0017 (12)	−0.0010 (11)
C4	0.0218 (17)	0.0199 (14)	0.0178 (14)	0.0021 (12)	0.0021 (12)	−0.0021 (11)
C5	0.0260 (19)	0.0249 (16)	0.0215 (16)	−0.0050 (13)	0.0023 (13)	−0.0017 (12)
C6	0.0149 (16)	0.0374 (19)	0.0259 (16)	0.0000 (13)	−0.0005 (12)	−0.0001 (13)
C7	0.0201 (17)	0.0264 (16)	0.0241 (16)	0.0062 (13)	0.0019 (13)	0.0034 (12)
N1	0.0302 (16)	0.0175 (13)	0.0286 (15)	0.0028 (12)	0.0018 (12)	−0.0019 (11)
O1	0.0352 (14)	0.0188 (11)	0.0432 (15)	0.0014 (10)	−0.0021 (11)	0.0014 (10)
O2	0.0270 (13)	0.0249 (12)	0.0516 (15)	−0.0031 (10)	0.0005 (11)	−0.0095 (11)
O3	0.0301 (14)	0.0248 (13)	0.0699 (19)	0.0093 (12)	−0.0115 (13)	−0.0070 (13)
O4	0.0391 (16)	0.0209 (12)	0.0625 (18)	−0.0042 (11)	−0.0030 (13)	−0.0034 (12)
C8	0.0320 (19)	0.0172 (15)	0.0247 (15)	−0.0062 (13)	0.0027 (13)	−0.0059 (12)
C9	0.0243 (17)	0.0204 (15)	0.0195 (15)	−0.0018 (12)	0.0035 (13)	−0.0024 (11)
C10	0.0202 (17)	0.0229 (16)	0.0192 (15)	−0.0023 (13)	0.0003 (12)	−0.0037 (11)
C11	0.0275 (18)	0.0193 (15)	0.0175 (14)	−0.0003 (13)	0.0041 (12)	−0.0008 (12)
C12	0.0230 (18)	0.0285 (17)	0.0204 (15)	−0.0072 (13)	0.0025 (13)	−0.0034 (13)
C13	0.0222 (18)	0.0349 (19)	0.0251 (17)	−0.0019 (13)	−0.0001 (13)	−0.0003 (13)
C14	0.0314 (19)	0.0235 (16)	0.0249 (16)	0.0013 (13)	−0.0001 (14)	0.0005 (13)
N2	0.0353 (18)	0.0204 (13)	0.0274 (15)	−0.0017 (13)	−0.0003 (13)	−0.0049 (11)
O5	0.0388 (14)	0.0184 (11)	0.0454 (15)	−0.0015 (10)	−0.0008 (12)	−0.0011 (10)
O6	0.0296 (14)	0.0266 (13)	0.0529 (16)	−0.0034 (11)	−0.0054 (12)	−0.0146 (11)
O7	0.0380 (16)	0.0282 (14)	0.066 (2)	0.0069 (13)	−0.0156 (14)	−0.0071 (13)
O8	0.0461 (16)	0.0173 (11)	0.0493 (15)	−0.0075 (11)	0.0033 (13)	−0.0027 (11)
C15	0.037 (2)	0.0301 (16)	0.0328 (18)	0.0057 (15)	−0.0043 (15)	0.0010 (14)
C16	0.0286 (18)	0.0296 (17)	0.042 (2)	−0.0019 (14)	−0.0051 (15)	0.0037 (14)
C17	0.041 (2)	0.0240 (16)	0.0384 (19)	0.0009 (15)	−0.0174 (16)	−0.0036 (14)
C18	0.051 (2)	0.0314 (18)	0.0358 (19)	0.0082 (17)	0.0066 (17)	0.0024 (15)
C19	0.0296 (18)	0.0252 (16)	0.057 (2)	−0.0014 (14)	0.0043 (16)	0.0026 (16)
N3	0.0319 (16)	0.0193 (12)	0.0431 (17)	0.0009 (11)	−0.0152 (13)	−0.0053 (11)

### Geometric parameters (Å, º)

**Table:**

C1—C2	1.488 (4)	C10—C11	1.381 (4)
C1—O1	1.311 (4)	C11—C12	1.386 (5)
C1—O2	1.214 (4)	C11—N2	1.467 (4)
C2—C3	1.398 (4)	C12—H12	0.9500
C2—C7	1.397 (4)	C12—C13	1.371 (5)
C3—H3	0.9500	C13—H13	0.9500
C3—C4	1.380 (4)	C13—C14	1.393 (5)
C4—C5	1.382 (4)	C14—H14	0.9500
C4—N1	1.472 (4)	N2—O7	1.217 (4)
C5—H5	0.9500	N2—O8	1.234 (4)
C5—C6	1.395 (4)	C15—H15	0.9500
C6—H6	0.9500	C15—C16	1.366 (5)
C6—C7	1.378 (5)	C15—N3	1.327 (5)
C7—H7	0.9500	C16—H16	0.9500
N1—O3	1.223 (4)	C16—C17	1.374 (6)
N1—O4	1.213 (4)	C17—H17	0.9500
O1—H1	0.83 (4)	C17—C18	1.379 (6)
C8—C9	1.520 (4)	C18—H18	0.9500
C8—O5	1.260 (4)	C18—C19	1.366 (6)
C8—O6	1.242 (4)	C19—H19	0.9500
C9—C10	1.378 (4)	C19—N3	1.331 (5)
C9—C14	1.389 (5)	N3—H3A	0.88 (4)
C10—H10	0.9500		
			
O1—C1—C2	113.5 (3)	C10—C11—C12	122.9 (3)
O2—C1—C2	121.8 (3)	C10—C11—N2	117.6 (3)
O2—C1—O1	124.7 (3)	C12—C11—N2	119.5 (3)
C3—C2—C1	117.6 (3)	C11—C12—H12	121.2
C7—C2—C1	123.4 (3)	C13—C12—C11	117.7 (3)
C7—C2—C3	118.9 (3)	C13—C12—H12	121.2
C2—C3—H3	120.4	C12—C13—H13	119.6
C4—C3—C2	119.1 (3)	C12—C13—C14	120.9 (3)
C4—C3—H3	120.4	C14—C13—H13	119.6
C3—C4—C5	122.7 (3)	C9—C14—C13	120.1 (3)
C3—C4—N1	118.3 (3)	C9—C14—H14	119.9
C5—C4—N1	118.9 (3)	C13—C14—H14	119.9
C4—C5—H5	121.2	O7—N2—C11	118.6 (3)
C4—C5—C6	117.6 (3)	O7—N2—O8	123.4 (3)
C6—C5—H5	121.2	O8—N2—C11	117.9 (3)
C5—C6—H6	119.5	C16—C15—H15	119.9
C7—C6—C5	121.0 (3)	N3—C15—H15	119.9
C7—C6—H6	119.5	N3—C15—C16	120.1 (4)
C2—C7—H7	119.7	C15—C16—H16	120.5
C6—C7—C2	120.6 (3)	C15—C16—C17	119.1 (4)
C6—C7—H7	119.7	C17—C16—H16	120.5
O3—N1—C4	117.8 (3)	C16—C17—H17	120.1
O4—N1—C4	118.5 (3)	C16—C17—C18	119.9 (3)
O4—N1—O3	123.7 (3)	C18—C17—H17	120.1
C1—O1—H1	105 (3)	C17—C18—H18	120.7
O5—C8—C9	116.6 (3)	C19—C18—C17	118.6 (4)
O6—C8—C9	117.3 (3)	C19—C18—H18	120.7
O6—C8—O5	126.2 (3)	C18—C19—H19	119.8
C10—C9—C8	119.2 (3)	N3—C19—C18	120.4 (4)
C10—C9—C14	119.8 (3)	N3—C19—H19	119.8
C14—C9—C8	120.9 (3)	C15—N3—C19	121.9 (3)
C9—C10—H10	120.7	C15—N3—H3A	113 (3)
C9—C10—C11	118.6 (3)	C19—N3—H3A	124 (3)
C11—C10—H10	120.7		
			
C1—C2—C3—C4	179.4 (3)	C9—C10—C11—N2	179.6 (3)
C1—C2—C7—C6	−179.1 (3)	C10—C9—C14—C13	0.1 (5)
C2—C3—C4—C5	−0.4 (5)	C10—C11—C12—C13	1.1 (5)
C2—C3—C4—N1	−179.6 (3)	C10—C11—N2—O7	−4.4 (5)
C3—C2—C7—C6	0.0 (5)	C10—C11—N2—O8	175.5 (3)
C3—C4—C5—C6	0.2 (5)	C11—C12—C13—C14	−0.9 (5)
C3—C4—N1—O3	2.8 (4)	C12—C11—N2—O7	175.8 (3)
C3—C4—N1—O4	−177.3 (3)	C12—C11—N2—O8	−4.2 (4)
C4—C5—C6—C7	0.1 (5)	C12—C13—C14—C9	0.4 (5)
C5—C4—N1—O3	−176.4 (3)	C14—C9—C10—C11	0.0 (5)
C5—C4—N1—O4	3.5 (4)	N2—C11—C12—C13	−179.2 (3)
C5—C6—C7—C2	−0.2 (5)	O5—C8—C9—C10	179.5 (3)
C7—C2—C3—C4	0.3 (4)	O5—C8—C9—C14	−2.1 (5)
N1—C4—C5—C6	179.4 (3)	O6—C8—C9—C10	−1.7 (5)
O1—C1—C2—C3	175.3 (3)	O6—C8—C9—C14	176.7 (3)
O1—C1—C2—C7	−5.6 (4)	C15—C16—C17—C18	−0.7 (5)
O2—C1—C2—C3	−5.1 (5)	C16—C15—N3—C19	1.1 (5)
O2—C1—C2—C7	174.0 (3)	C16—C17—C18—C19	0.8 (5)
C8—C9—C10—C11	178.4 (3)	C17—C18—C19—N3	0.0 (5)
C8—C9—C14—C13	−178.3 (3)	C18—C19—N3—C15	−1.0 (5)
C9—C10—C11—C12	−0.6 (5)	N3—C15—C16—C17	−0.2 (5)

### Hydrogen-bond geometry (Å, º)

**Table:**

*D*—H···*A*	*D*—H	H···*A*	*D*···*A*	*D*—H···*A*
O1—H1···O5	0.83 (4)	1.69 (4)	2.508 (3)	169 (5)
N3—H3*A*···O6	0.88 (4)	1.81 (4)	2.667 (4)	164 (4)
